# Placental Hypomethylation Is More Pronounced in Genomic Loci Devoid of Retroelements

**DOI:** 10.1534/g3.116.030379

**Published:** 2016-04-27

**Authors:** Aniruddha Chatterjee, Erin C. Macaulay, Euan J. Rodger, Peter A. Stockwell, Matthew F. Parry, Hester E. Roberts, Tania L. Slatter, Noelyn A. Hung, Celia J. Devenish, Ian M. Morison

**Affiliations:** * Department of Pathology, Dunedin School of Medicine, University of Otago, Dunedin 9054, New Zealand; † Gravida: National Centre for Growth and Development, University of Auckland, 1142, New Zealand; ‡ Maurice Wilkins Centre for Molecular Biodiscovery, Auckland 1010, New Zealand; § Department of Biochemistry, University of Otago, Dunedin 9054, New Zealand; ** Department of Mathematics and Statistics, University of Otago, Dunedin 9054, New Zealand; †† Department of Women’s and Children’s Health, Dunedin School of Medicine, University of Otago, Dunedin 9054, New Zealand

**Keywords:** hypomethylation, placenta, neutrophil, retroelement, reduced representation bisulfite sequencing

## Abstract

The human placenta is hypomethylated compared to somatic tissues. However, the degree and specificity of placental hypomethylation across the genome is unclear. We assessed genome-wide methylation of the human placenta and compared it to that of the neutrophil, a representative homogeneous somatic cell. We observed global hypomethylation in placenta (relative reduction of 22%) compared to neutrophils. Placental hypomethylation was pronounced in intergenic regions and gene bodies, while the unmethylated state of the promoter remained conserved in both tissues. For every class of repeat elements, the placenta showed lower methylation but the degree of hypomethylation differed substantially between these classes. However, some retroelements, especially the evolutionarily younger Alu elements, retained high levels of placental methylation. Surprisingly, nonretrotransposon-containing sequences showed a greater degree of placental hypomethylation than retrotransposons in every genomic element (intergenic, introns, and exons) except promoters. The differentially methylated fragments (DMFs) in placenta and neutrophils were enriched in gene-poor and CpG-poor regions. The placentally hypomethylated DMFs were enriched in genomic regions that are usually inactive, whereas hypermethylated DMFs were enriched in active regions. Hypomethylation of the human placenta is not specific to retroelements, indicating that the evolutionary advantages of placental hypomethylation go beyond those provided by expression of retrotransposons and retrogenes.

DNA methylation plays a role in several biological processes including lineage specification, chromosome X inactivation, genomic imprinting, maintenance of genomic stability, and retrotransposon silencing (Bird 2002; Chatterjee and Eccles 2015). In comparison to somatic tissues, all studied mammalian placentas are hypomethylated, suggesting conservation of a functional role (Schroeder *et al.* 2015). Human placenta has been reported to have 14–25% lower levels of global DNA methylation than somatic tissues (Ehrlich *et al.* 1982; Tsien *et al.* 2002; Fuke *et al.* 2004; Novakovic *et al.* 2010; Schroeder *et al.* 2013) (Supplemental Material, Table S1). Early analyses of specific genomic elements focused on repetitive satellite and Alu DNA that were hypomethylated in the mouse placenta (Chapman *et al.* 1984; Hellmann-Blumberg *et al.* 1993). In addition, the methylation of a consensus LINE1 sequence was reduced by approximately 43% compared to blood (Cotton *et al.* 2009). At three specific LTR-derived gene promoters, an 80% reduction in methylation was observed, whereas LTRs from random human endogenous retroviral sequences showed 11–14% reduction in methylation (Reiss *et al.* 2007). Therefore, LTR methylation appears to be context-dependent but relatively retained in the placenta. In addition, we have shown marked hypomethylation of the SINE-derived promoter of *KCNH5* and the LTR-derived promoters of *INSL4*, *ERVWE1*, *EDNRB*, *PTN*, and *MID1* in placenta compared to somatic tissues (Macaulay *et al.* 2011). However, there is no detailed documentation of genome-wide placental methylation with respect to specific genomic elements.

Placental-specific epigenetic modification, such as DNA hypomethylation, is hypothesized to support the unique functions of the placenta (Reiss *et al.* 2007; Macaulay *et al.* 2011). Activation of retrotransposon-derived genes in the placenta is associated with hypomethylation, and has been well documented (Reiss *et al.* 2007; Cohen *et al.* 2011; Macaulay *et al.* 2011). These genes play an essential role in human placental function through a variety of candidate mechanisms including trophoblast syncytial formation (Frendo *et al.* 2003; Dupressoir *et al.* 2012) and immunosuppression (Schlecht-Louf *et al.* 2010), and they have been proposed as the original selective driving force for global hypomethylation of the placenta (Hemberger 2010). Therefore, we hypothesized that hypomethylation would be relatively specific for retrotransposons and retrogenes.

In this study, we used reduced representation bisulfite sequencing (RRBS) to quantify genome-wide methylation of human placentas and compared their methylation profiles with those of a homogeneous somatic cell type, neutrophils. Although RRBS covers a small proportion of the genome, we provide a high coverage of the analyzed regions, thereby permitting firm conclusions from a representative portion of the genome. Further, RRBS covers genomic regions that are likely to have functional consequence and, therefore, this analysis provides insight into the genome regulation of placenta. We investigated major classes of genomic elements and determined their contribution to global hypomethylation of the placenta. Further, we examined regions that were significantly differentially methylated between placenta and neutrophils to gain insight into the potential role of these regions in placental genome function.

## Materials and Methods

### Placentas

Placentas, ranging in gestational age from 24–40 wk, were collected by the Otago Placental Study (University of Otago, Dunedin). Collection was approved by the Lower South Regional Ethics Committee (LRS/09/09/038). They are described in Table S2. For this study, a 0.5 cm^3^ piece of tissue was dissected from the center of a transmural section of placenta. To minimize contamination from maternal blood, samples were gently disrupted and washed and rinsed in phosphate buffered saline (PBS).

### Neutrophils

Collection of neutrophils was approved by the Multi-region Ethics Committee (MEC/09/07/068). EDTA-anticoagulated blood from 11 healthy individuals aged from 26–34 yr (median = 31 yr; five male and six female) was diluted (1:1) in PBS, layered on Ficoll-Paque PLUS (GE Healthcare), and centrifuged at 400 × *g* for 40 min at room temperature. The pellet (neutrophils and red cells) was lysed with 0.17 M NH_4_Cl, centrifuged at 300 × *g* for 10 min, and resuspended in PBS. All samples contained > 90% neutrophils (median purity = 96%).

### DNA extraction

Placental and neutrophil DNA was extracted using the QIAamp DNA mini kit (Qiagen) following the manufacturer’s protocol, except that the proteinase K treatment was performed overnight at 55°.

### RRBS library preparation and sequencing

RRBS libraries were prepared following our previously published protocols (Chatterjee *et al.* 2012a; 2012b, 2013, 2014). Briefly, 2.5 µg genomic DNA was digested overnight with *Msp*I (New England Biolabs, Ipswich, MA), The digested fragment was end-repaired, a 3′ A-overhang was added, and the methylated adaptors (Illumina, San Diego, CA) were ligated. Next, 40–220 bp (preadaptor ligation size) fragments were size-selected from 3% Nusieve agarose gels (Lonza, Basel, Switzerland) and subsequently bisulfite-converted with EZ DNA methylation kit (Zymo Research, Irvine, CA). Bisulfite-converted libraries were amplified by PCR and a second round of size selection was performed. The RRBS libraries were assessed on a 2100 Bioanalyzer (Agilent Technologies) using the high sensitivity DNA chip. A Qubit fluorometer (Life Technologies) was used for quantification of DNA in the libraries. The RRBS libraries were sequenced (single-ended 100 bp) in an Illumina HiSeq2000. Neutrophil methylomes yielded a total of 344 million sequence reads, whereas placental methylomes yielded a total of 153 million sequence reads.

### DNA methylation analysis

Quality checks, removal of adaptor sequences, and quality-based hard-trimming were performed as previously described (Chatterjee *et al.* 2012b, 2013). Bismark v0.6.4 (Krueger and Andrews 2011) was used to align the processed sequence reads to the reference genome (GRCh37). We applied stringent mapping criteria by allowing only one mismatch (default = 2) in the seed (*i.e.*, in the first 28 bp of the sequenced reads). After filtering for low quality sequences, a median of 67% and 65% unique alignments were obtained for placenta and neutrophil RRBS libraries, respectively. The median non-CpG DNA methylation was 1.95% and 2.45% in the neutrophil and placental libraries, respectively (as measured by Bismark alignment), indicating effective bisulfite conversion and low levels of true non-CpG methylation.

Differential methylation analysis was performed using our in-house Differential Methylation Analysis Package (DMAP) (Stockwell *et al.* 2014), which contains two main programs (diffmeth and identgenloc). *Msp*I fragments were used as the unit of analysis for DNA methylation. Each sample was filtered for fragments that had at least two CpG sites covered by 10 or more sequenced reads (F2 t10 switch in the diffmeth program of DMAP tool). Then the common fragments, where at least six individuals from both tissues types met the F2 t10 criteria, were selected. An F test (ANOVA) was performed on these common fragments to test for significant differences between placenta and neutrophil fragment methylation. Initially, 7158 fragments passed an adjusted *P* value cut-off (see statistical analysis). We then selected those that had ≥ 25% methylation difference between the two groups, giving 6767 fragments that were classified as differentially methylated fragments (DMFs). The analysis was performed using a Mac Pro with 64-bit duo quad core Intel Xeon processors, and with 22 GB RAM running MacOS 10.6.

### Overlap analysis with chromatin segmentation, histone modification, and transcription factors

Genomic and epigenomic feature analysis of the DMFs was performed using Epiexplorer tool (Halachev *et al.* 2012). The genomic coordinates of the DMFs were uploaded to the Epiexplorer server. The hypo- and hypermethylated DMFs were compared with the 25,392 fragments that were not differentially methylated (non-DMFs). The fragments that showed a strong overlap (*i.e.*, ≥ 50% of a fragment overlapped with a feature) were selected and shown in this analysis. We performed “any tissue” analysis in the Epiexplorer platform. The “any tissue” analysis is the summation of all available data from nine different cell lines for a feature (the nine cell lines are GM12878, H1hESC, HepG2, HMEC, HSMM, HUVEC, K562, NHEK, and NHLF).

### Gene ontology analysis

Gene ontology (GO) term enrichment and functional annotation analyses were done using the Database for Annotation, Visualization, and Integrated Discovery (DAVID, v6.7) (Huang *et al.* 2009). Our gene sets were tested against the background of all protein-coding human genes.

### Statistical analysis

For detection of DMFs, an F test was performed using DMAP (Stockwell *et al.* 2014) To control for family-wise error rate, a Bonferroni correction was applied. Starting from a *P* value cut-off of 0.05, Bonferroni correction resulted in an adjusted P value cut-off of 1.55 × 10^−6^. For Gene–term enrichment analysis, *P* values were calculated with a modified Fisher’s exact test and *P* < 0.05 was considered significant. The enrichment score in the gene-ontology analysis was the geometric mean of all the enrichment *P* values for each annotation term associated with the gene members in the group. The hypergeometric test was used to assess overlap with PMDs. This test calculates the probability of drawing a specific number of successes (from a total number of draw) from a population. The other statistical tests described in this article (*e.g.*, chromosome-wide distribution, calculation of overlap with regulatory feature) were performed using a chi-square test with Yates correction and *P* < 0.05 was considered significant.

### Data availability

The DNA methylation data generated for placentas and neutrophils have been submitted to the NCBI Gene Expression Omnibus. The neutrophil RRBS data are available in accession number GSE59163 and the placenta methylomes are available under accession number GSE59988.

## Results

### Placenta and neutrophil DNA methylomes

RRBS was used to generate DNA methylomes of 11 human placentas (Table S2). Methylomes of purified neutrophils (a homogeneous somatic tissue) from 11 healthy individuals (five male and six female) aged 26–34 yr were similarly obtained (Chatterjee *et al.* 2015). The distribution and level of CpG DNA methylation (on a scale of 0–1) was determined, using *Msp*I fragments (40–220 bp) as the unit of analysis rather than individual CpG sites or a tiled window approach, as previously explained (Stockwell *et al.* 2014; Chatterjee *et al.* 2015, 2016). 279,762 *Msp*I fragments fulfilled inclusion criteria in at least one placenta and one neutrophil sample (10 or more reads at ≥ 2 CpG sites). 272,390 of these sites were autosomal and 7105 were from chromosome X. These are referred to as “total analyzed fragments.” For comparisons between placentas and neutrophils, we required that each fragment fulfilled the coverage criteria in at least six individuals from each group. These fragments are subsequently referred to as “comparison fragments” and comprised 32,163 fragments (31,304 from autosomes, 853 from chromosome X, and 7 from chromosome Y) that covered a total of 226,672 CpG sites. Considering the number of fragments that fulfilled the inclusion criteria, and the number of male and female samples in this study, we chose to refrain from specific chromosome X analysis. The global CpG methylation in the RRBS fraction of the human placenta was calculated by taking the weighted mean of the 32,163 fragments, based on the number of CpG sites in each fragment. The average methylation of the 32,163 fragments was 0.371 in the placenta and 0.475 in the neutrophils. Since RRBS enriches for CpG islands, which are often unmethylated, measurements of methylation by RRBS are lower than that of the whole-genome.

### Distribution of methylation in placenta and neutrophils

**Figure 1:**
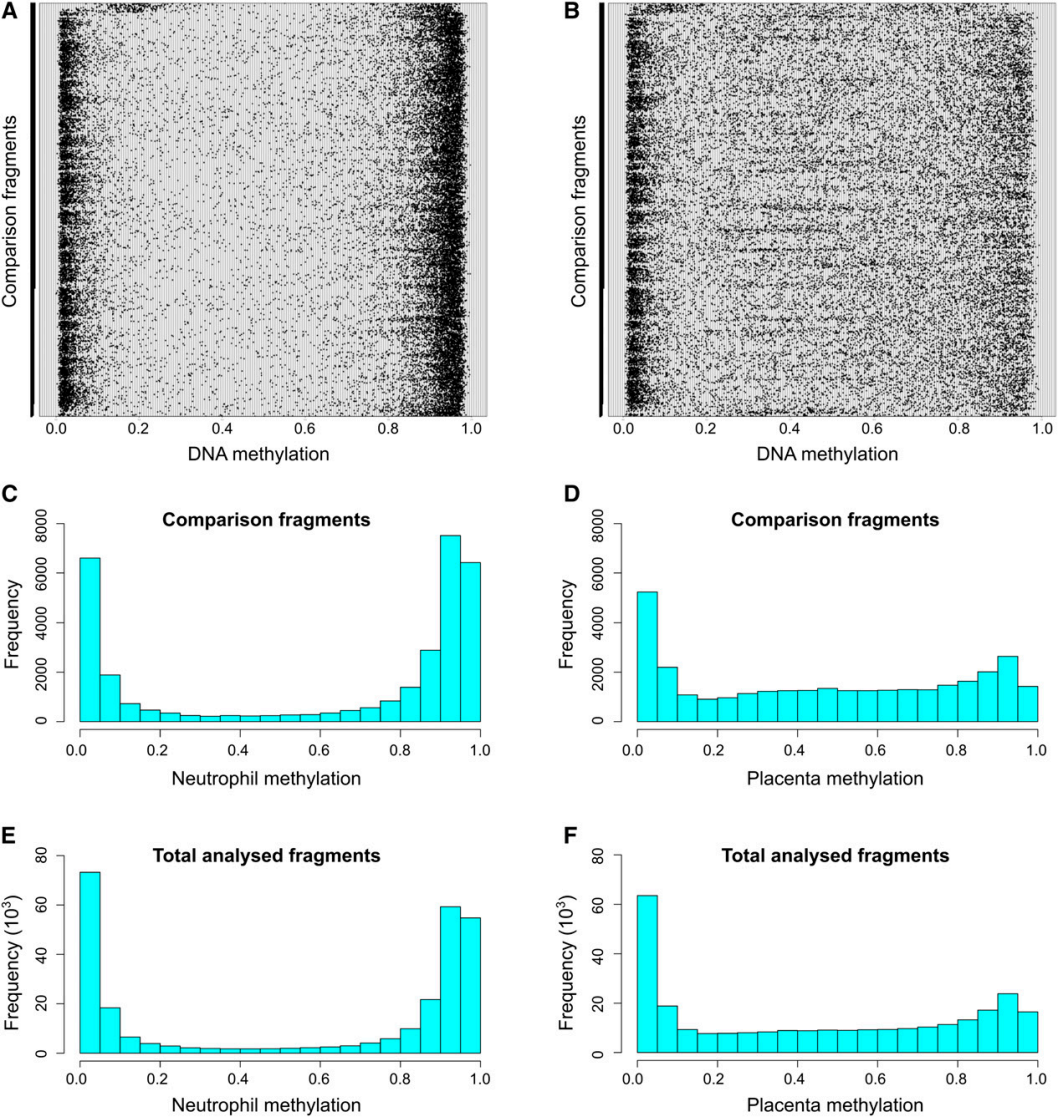
Methylation in placentas and neutrophils. The mean methylation of each of 32,163 comparison fragments from chromosome 1 (bottom) to chromosome X in neutrophils (A) and placenta (B). (C) and (D) Histograms of the data shown in (A) and (B). (E) and (F) Histograms of 279,762 *Msp*I fragments (total analyzed fragments).

Neutrophils showed a bimodal methylation pattern, in that 76% of the neutrophil fragments were highly methylated (≥ 0.70) and 13.4% of the fragments had low methylation (≤ 0.30) (Figure 1, A and B), similar to previous reports for somatic tissues (Meissner *et al.* 2008). The placenta, however, showed a different distribution of methylation, with almost equal proportions of fragments with low, high, or intermediate (> 0.30 and < 0.70) methylation (Figure 1, C and D).

To determine whether the different distribution of methylation observed in neutrophils and placentas might have been biased in the selection of the comparison fragments, we plotted similar histograms for the total analyzed fragments (Figure 1, E and F, Figure S1, and Figure S2). The distributions of the comparison and total analyzed fragments were similar. Apart from chromosome X, the distribution of methylation in neutrophils and in placentas was similar in all chromosomes (Figure S3 and Figure S4). However, as expected, in both tissues, regions with higher CpG density tend to be less methylated (Figure S5 and Figure S6).

**Figure 2:**
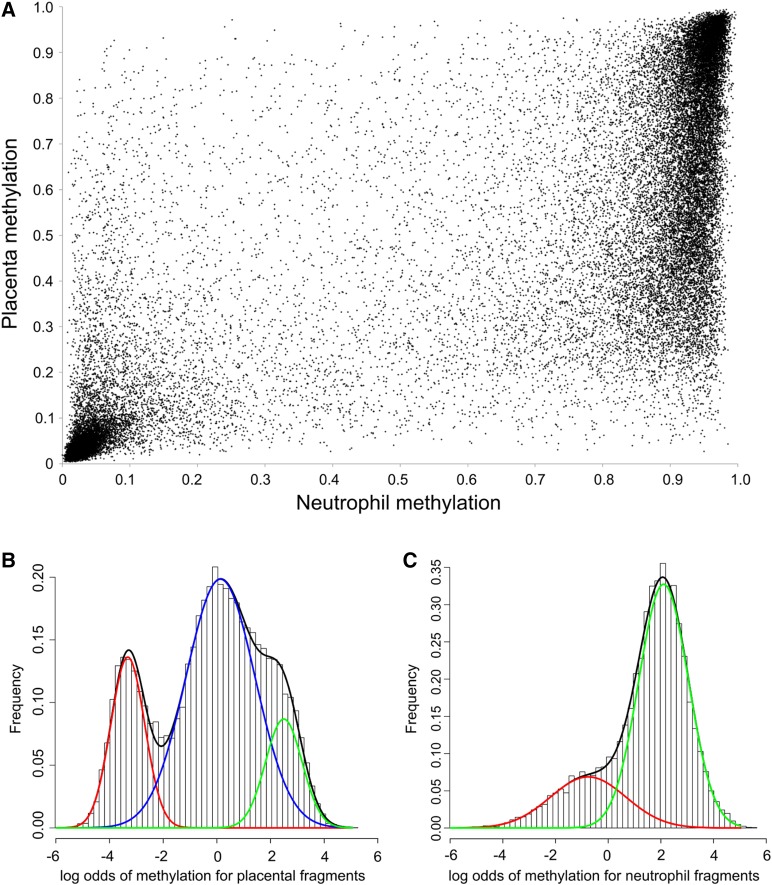
Methylation distribution in placentas and neutrophils. (A) Mean methylation of comparison fragments for neutrophils (x-axis) and placentas (y-axis). (B) and (C) Logit transformation of methylation values. The black curves show the total density; the red, blue, and green curves show the mathematical decomposition of the distribution of DNA methylation into their Gaussian components.

Comparison of the methylation in placenta and neutrophils demonstrated that the placental fragments with intermediate methylation were predominantly highly methylated in neutrophils (Figure 2A). For improved visualization of the distribution of DNA methylation, and to resolve the contributing Gaussian components, we used the logit function to transform the data to log odds values to provide better resolution of low and high methylation (Benaglia *et al.* 2009). The methylation of the placenta could be decomposed into three Gaussian components having low, intermediate, and high methylation (comprising 21.5%, 63.8%, and 14.7% of fragments, respectively) with mean log odds values of −3.34, 0.10, and 2.45 (corresponding to methylation levels of 0.034, 0.525, and 0.920) (Figure 2B). The distribution of log odds of neutrophil methylation cluster, is best described as a mixture of two Gaussian components with predominant contributions from the low and highly methylated fragments (Figure 2C).

### Methylation distribution in different genomic elements

**Figure 3:**
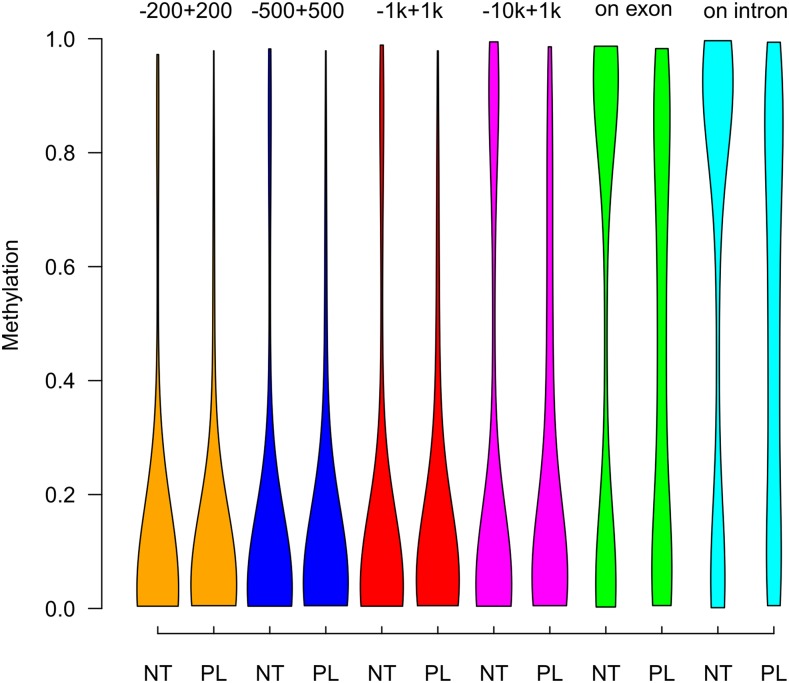
Violin plots of neutrophil and placental methylation in promoters, exons, and introns. + and – denote fragment distances from the start of the gene in bp or kb (k). Plots have equal area. NT, neutrophils; PL, placenta.

We then compared methylation in different genomic windows around transcription start sites (TSS) and genes (Figure 3). In both tissues, regions around the TSS (200 bp, 500 bp, or 1 kb either side) were almost completely unmethylated. For exons and introns, the comparison fragments showed either low (< 0.30) or high (> 0.70) methylation in neutrophils, with a larger proportion being highly methylated (median methylation of 0.71 and 0.72 in exons and introns, respectively). On the other hand, placentas had fewer highly methylated fragments and a consequent increase in intermediately methylated fragments (median methylation of 0.59 and 0.58 in exons and introns, respectively) (Figure 3 and Table S3).

### Comparison of methylation in retrotransposed elements

**Figure 4:**
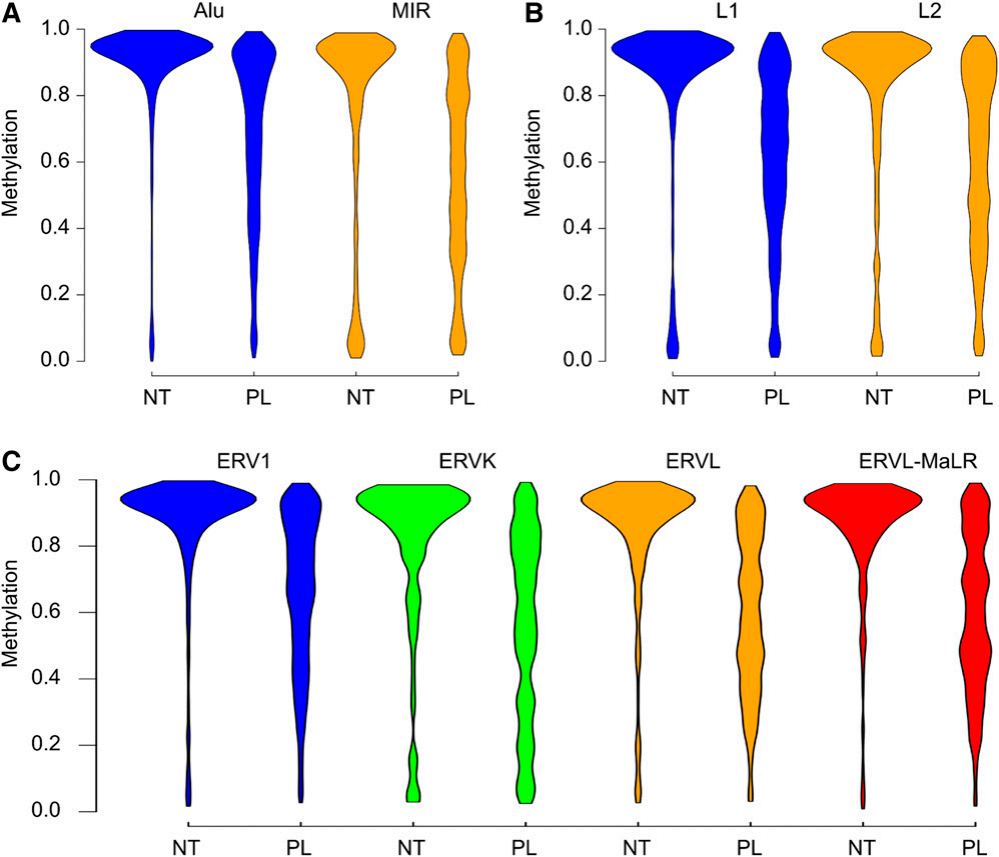
Violin plots of neutrophil and placental methylation for different classes of retrotransposons. (A) SINE elements (Alu and MIR), (B) LINE elements (L1 and L2), and (C) LTRs (ERV1, ERVK, ERVL, and ERVL-MaLR). Plots have equal area. NT, neutrophils; PL, placenta.

To test whether the placenta shows specific hypomethylation of retrotransposons, we compared the methylation of RRBS fragments that overlapped SINEs (Alu and MIR class), LINEs (L1 and L2), and LTRs (ERV1, ERVK, ERVL, and ERVL-MaLR). As expected, neutrophils were heavily methylated in every class of repeat elements analyzed (Yoder *et al.* 1997; Bird 2002) (Figure 4, A–C).

Although most repeat families showed relative hypomethylation in the placenta, the difference varied between subfamilies. In the SINE family, Alu elements, which are evolutionarily younger [integration of different classes of Alu repeats occurred between 5–55 million years ago (mya) (Batzer and Deininger 2002)] showed less difference in methylation (median methylation 0.94 and 0.76 in neutrophils and placenta, respectively) compared to the mammalian-wide interspersed repeats [MIRs, integration in human genome took place ∼130 mya (Bourque *et al.* 2008)] that showed median methylation of 0.89 and 0.58, respectively (Figure 4A). In contrast, in the LTR family, the ERV1 subclass, which is evolutionarily old (56–81 mya), showed less difference in methylation (median methylation 0.93 and 0.71 in neutrophils and placenta, respectively) than the evolutionarily younger family members such as ERVK (expansion age between 32–53 mya (Sverdlov 2000; Bourque *et al.* 2008)) (median methylation 0.90 and 0.59, respectively) (Figure 4C). The methylation of fragments containing ERVL (median methylation 0.91 and 0.61) and ERVL-MaLR (median methylation 0.92 and 0.62) elements also showed a relatively large difference between placenta and neutrophils (Figure 4C and Table S4). For LINE elements, the evolutionarily younger L1 elements showed the same degree (median methylation 0.92 and 0.65) of placental hypomethylation as the older L2 elements (median methylation 0.91 and 0.64) (Figure 4B).

### Methylation in retrotransposon *vs.* nonretrotransposon-containing fragments

Next, we asked whether the hypomethylation in placenta is specific to retrotransposons. Satellite sequence-containing fragments showed strikingly lower methylation in placenta (median methylation 0.88 and 0.53 in neutrophils and placenta respectively). In contrast, low complexity and simple repeat classes were largely unmethylated in both placenta and neutrophils (Table S4).

**Figure 5:**
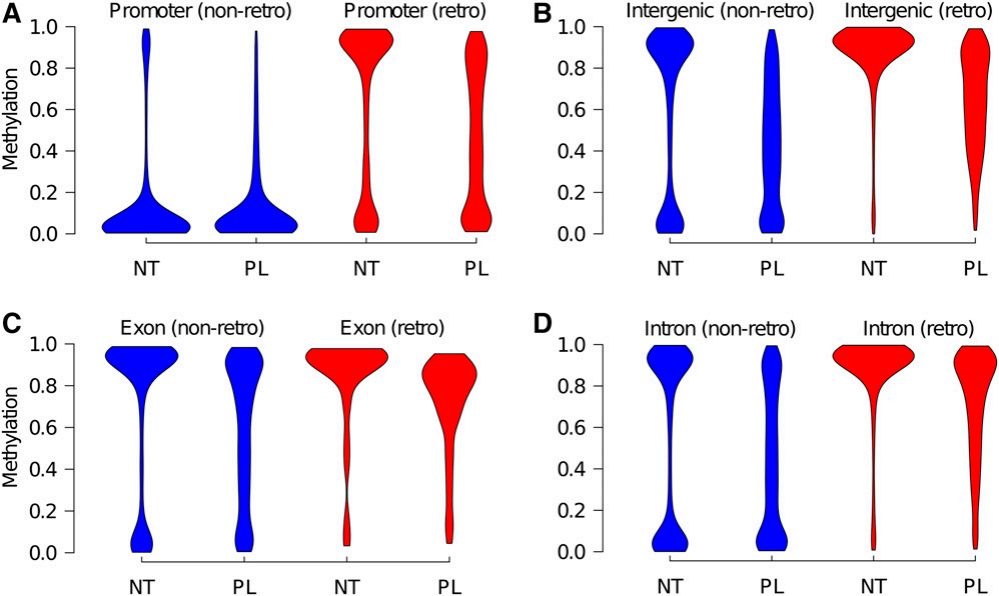
Methylation of retroelement and nonretroelement-containing fragments in neutrophils and placental. (A) promoters, (B) intergenic regions, (C) exons, and (D) introns. Plots have equal area. NT, neutrophils; PL, placenta.

The four major genomic regions (promoter, intergenic, exons, and introns) were then separated into retroelement- and nonretroelement-containing fragments and the methylation was analyzed for each group (Figure 5, A–D and Table S5).

Intergenic fragments (> 5 kb from TSS) were the largest group in our analysis (n = 12,040). Within the intergenic group, fragments overlapping retroelements (n = 4664) were highly methylated in neutrophils (median = 0.93), whereas the methylation in placenta (median = 0.66) was 0.27 lower (Figure 5B and Table S5). In the nonretroelement fragments (n = 7376), the reduction in placental methylation was even greater. The median methylation for neutrophils and placenta in nonretroelement fragments was 0.82 and 0.42, respectively (*i.e.*, median reduction = 0.40).

**Figure 6:**
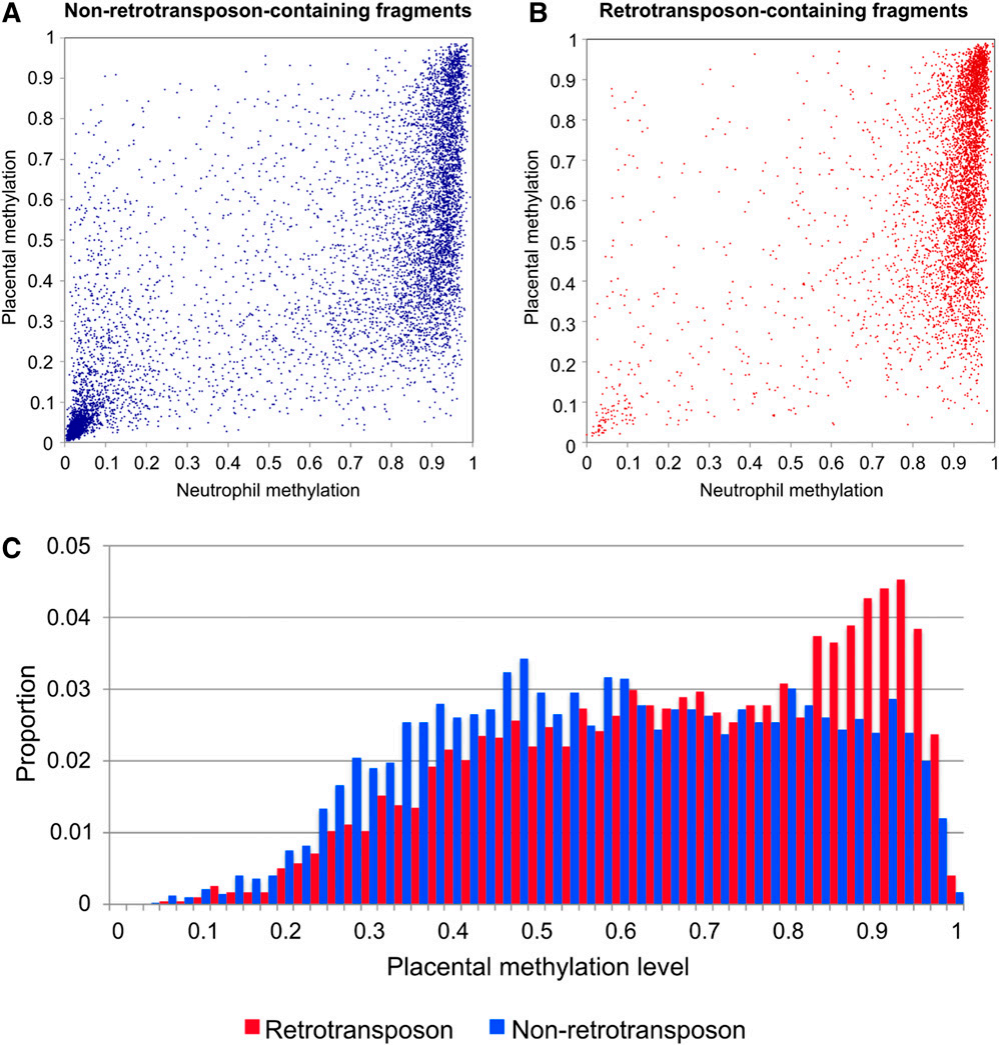
Comparison of methylation of retro and nonretroelement-containing fragments in intergenic regions. (A) Comparison of neutrophil and placental methylation of intergenic fragments not containing retroelements, (B) comparison of neutrophil and placental methylation of intergenic retroelement-containing fragments, and (C) distribution of methylation in placenta of fragments having > 0.7 methylation in neutrophils.

To determine whether retrotransposons and nonretrotransposons are differently modified in the placenta, we first selected the largest group of fragments (*i.e.*, intergenic) and compared their methylation (Figure 6, A–B). Nonretrotransposon fragments were generally less methylated in placenta than retrotransposons. The group of fragments that were highly methylated in neutrophils (> 0.7) were selected, and the placental methylation of retrotransposon and nonretrotransposons were compared (Figure 6C). A disproportionate number of retrotransposons maintain high methylation in the placenta, whereas nonretrotransposons make up a disproportionate number of placenta fragments showing marked hypomethylation. We examined whether any particular class of retrotransposons contributes disproportionately to those that maintain the highest levels of methylation in the placenta (> 0.8). Alu elements were overrepresented, comprising 56% of the retrotransposons showing high methylation and 45% of those with < 0.8 methylation (Table S6). High levels of placental methylation (> 0.8) were retained by 39% of Alu-containing and 36% of ERV1-containing fragments, whereas only 21–24% of L1, MIR, ERVK, ERVL, and ERV-MaLR-containing elements retained high methylation.

Similarly, in introns, the difference in methylation between neutrophils and placenta was strikingly higher for nonretroelement fragments than for retroelement fragments (median difference = 0.38 and 0.14, respectively; Figure 5, A–D and Table S5). Exons also showed a similar trend, with a higher reduction of methylation in nonretroelements compared to retroelements (Table S5). The majority of promoter fragments did not contain retroelements and were predominantly unmethylated, but the 11% of retrotransposon-containing promoter fragments showed a reduction in placental methylation similar to that of other genomic regions. Taken together, these results demonstrate that, although retroelements are hypomethylated in placenta, the degree of hypomethylation is globally more pronounced in nonretroelements.

### Characterization of differentially methylated fragments

**Figure 7:**
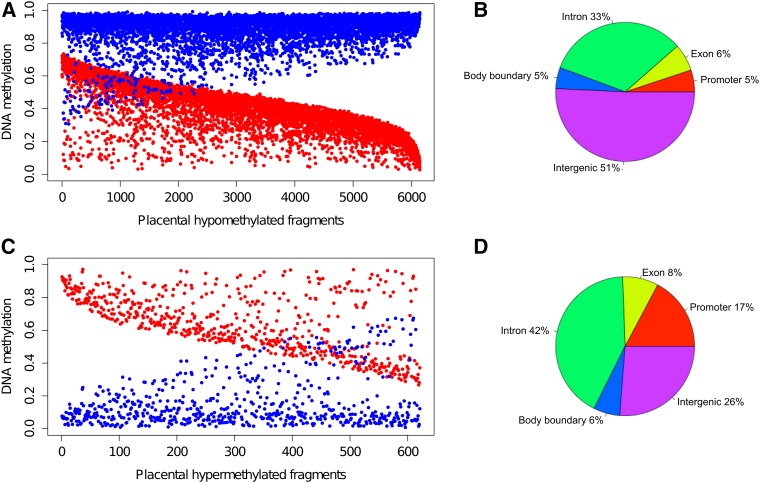
DNA methylation of differentially methylated fragments (DMFs). (A) placentally hypomethylated DMFs. (B) genomic distribution of the placentally hypomethylated DMFs. (C) placentally hypermethylated DMFs. (D) genomic distribution of the placentally hypermethylated DMFs. Mean methylation of fragments (mean methylation of individuals in one group) in the placenta and neutrophils are shown in red and blue, respectively.

An alternative approach was used to characterize regions with the largest methylation difference between placentas and neutrophils. Using a stringent statistical cut-off (analysis of variance followed by Bonferroni correction: *P* = 1.55 × 10^−6^), along with the requirement for an absolute difference between placental and neutrophil methylation of ≥ 0.25, we identified 6767 DMFs among the comparison fragments (see File S1 and Table S7). Of the DMFs, 90.8% were hypomethylated in placenta compared to neutrophils (6126 hypomethylated; 621 hypermethylated). The majority of DMFs had intermediate levels of methylation (0.30–0.70) in placenta (*i.e.*, 72% of the hypomethylated and 60% of the hypermethylated DMFs) (Figure 7, A and C). Of the comparison fragments, 12,430 fragments (38.6%) showed < 0.05 difference in the methylation, and 97.6% of these showed either high or low methylation in both placenta and neutrophils.

Annotation of the distribution of DMFs within genomic elements showed that placentally hypomethylated DMFs were predominantly distributed in intergenic regions (> 5 kb upstream; 51%) and in gene bodies (44%). Five percent of the placentally hypomethylated DMFs were in gene promoters (defined as −5–+1 kb from the TSS). On the other hand, 17% of placental hypermethylated DMFs were located in gene promoters (17%) (Figure 7, B and D).

The relationship of the DMFs with gene density and CpG density was then examined. The genome was divided into 1 Mb tiled windows, and each was assigned a density score by dividing the number of DMFs by the number of comparison fragments in a window (windows with < 3 comparison fragments were excluded). The tiles with high DMF density scores were more prevalent in gene-poor regions for both hypo- and hypermethylated DMFs (Pearson’s r = −0.35 and −0.36, respectively) (Figure S7 and Figure S8). Additionally, relatively CpG-poor regions were associated with high DMF density (Pearson’s r = −0.37 and −0.50 for hypo- and hypermethylated DMFs, respectively; Figure S9 and Figure S10).

Previously, Schroeder *et al.* (2013) reported that 37% of the placental genome consisted of partially methylated domains (PMDs) (7746 domains associated with 881 genes). The hypomethylated DMFs identified in this study overlapped with the PMDs (Table S8) and were significantly more prevalent in PMD regions compared to the nonhypomethylated fragments (37% compared to 25%, *P* value < 10^−16^, hypergeometric test).

### Role of placental DMFs in genome regulation

**Figure 8:**
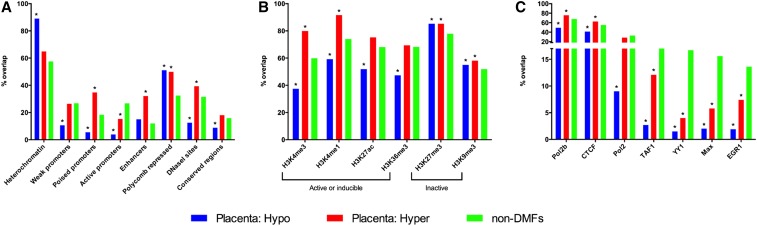
Overlap of placental differentially methylated fragments (DMFs) with genome regulation features. Compared to nonvariable fragments (non-DMFs), placental DMFs were analyzed for enrichment or depletion at (A) genomic regulatory features, (B) histone modifications, and (C) major transcription factor binding sites. Statistical significance was calculated using chi-square (with Yates correction), * *P* < 0.0001.

To investigate the potential role of the DMFs in genome regulation, we analyzed their overlap with chromatin segmentation features, histone modification marks, and transcription factor binding sites (Figure 8). Since the regulatory features of the placental and neutrophil genomes differ, and because curated ENCODE data were not available for these tissues, we used aggregated data from nine cell lines from the ENCODE project to obtain a representative “average” genomic state. Compared to non-DMFs, the placentally hypomethylated DMFs were significantly less abundant in promoters and DNase I hypersensitive sites (*P* < 0.0001, chi-square test); *i.e.*, transcriptionally active regions of the genome (Crawford *et al.* 2006). As a corollary, hypomethylated DMFs were more often associated with heterochromatin and weakly transcribed regions of the genome. In contrast, the placentally hypermethylated DMFs were significantly enriched at poised promoters, enhancers, and DNase I hypersensitive sites (Figure 8A).

The placentally hypomethylated DMFs showed significantly lower overlap than non-DMFs with active or inducible histone modification marks [H3K4me3, H3K4me1, H3K27ac, and H3K36me3 (Kimura 2013)], but were enriched for the repressive histone marks [H3K9me3 and H3K27me3 (Kimura 2013)] compared to non-DMFs. On the other hand, hypermethylated DMFs showed enrichment for active marks, H3K4me3 (enhancer mark) and H3K4me1, as well for repressive marks (Figure 8B). Compared to non-DMFs, hypo- and hypermethylated DMFs were significantly underrepresented at most of the transcription factor bindings sites analyzed. However, hypermethylated DMFs were enriched for Pol2b and CTCF binding sites (Figure 8C).

### Functional enrichment of differentially methylated genes in placenta

To assess the functions of differentially methylated genes, we performed a Gene Ontology (GO) enrichment analysis using genes that contained DMFs in their promoter regions (−5–+1 kb from TSS; 355 and 98 hypo- and hypermethylated genes, respectively). Hypomethylated genes in the placenta were enriched for epithelial structure maintenance, tissue homeostasis, digestion, and visual perception (Table S9). On the other hand, the hypermethylated genes were involved in regulation of transcription, gut morphogenesis, and mesoderm and blood vessel development (Table S10) (Schroeder *et al.* 2013).

## Discussion

The methylation of human placenta was compared to neutrophils at high resolution and showed hypomethylation (22%) of the placenta, in accordance with previous studies (Ehrlich *et al.* 1982; Tsien *et al.* 2002; Fuke *et al.* 2004; Novakovic *et al.* 2010; Schroeder *et al.* 2013). We found that hypomethylation affected every class of repeat elements, but that the degree of hypomethylation differed substantially between the repeat classes. Unexpectedly, the degree of placental hypomethylation was even greater in nonretrotransposon-containing sequences than in retrotransposons.

Each tissue type has its own specific methylation profile and some of the differences shown here may be specific to neutrophils. Neutrophils, however, provide a good surrogate for somatic tissue, because the methylation of neutrophils is similar to that of other blood cell types (Reinius *et al.* 2012) and the global methylation of blood cells is similar to that of other somatic tissues (Ehrlich *et al.* 1982; Fuke *et al.* 2004; Ziller *et al.* 2013; Schroeder *et al.* 2015). In addition, neutrophils were chosen as a representative somatic tissue since they are one of few readily available homogeneous somatic cell populations and they show stable methylation levels over the first three decades of life (Fuke *et al.* 2004). Furthermore, the differences between the placenta and neutrophils may be diluted by the presence of somatic cells (*e.g.*, vascular and immune cells) in the placenta (Wang and Zhao 2010). Future work could examine the methylation profiles of each the subpopulations of placental cells, particularly those of somatic (embryonic) *vs.* trophectoderm-derived (extraembryonic) cells.

The genomic distribution of hypomethylation in the placenta has previously been described in terms of PMDs (Schroeder *et al.* 2013). These are associated with repressive histone marks and regions with low gene content, and they also tend to show tissue-specific methylation patterns (Hansen *et al.* 2011; Plass *et al.* 2013). In this study, 19% of the placentally hypomethylated genes overlapped the PMD-containing genes, demonstrating that the majority of the hypomethylated sequences are located outside known PMDs.

Numerous hypotheses have been proposed for the evolutionary origin and roles of placental hypomethylation, and many of these relate to expression of retrotransposon-derived sequences. In this study, we show lower methylation at all classes of retrotransposons. The observed hypomethylation of LINEs is concordant with a previous study showing hypomethylation of a consensus LINE1 sequence in the placenta (Cotton *et al.* 2009). Interestingly, Alu elements maintain relatively high methylation in the placenta (19% lower in placenta than neutrophils). The methylation of placental Alu elements has previously been interpreted as the same as (Gama-Sosa *et al.* 1983), or lower than, somatic tissues (Hellmann-Blumberg *et al.* 1993). Interestingly, Alu elements showed less hypomethylation than the other class of SINEs, the MIRs, which had 35% less methylation than neutrophils. The higher methylation in evolutionarily younger families, such as Alu elements, could suggest that the repression of these families via methylation is necessary for placental function (Guo *et al.* 2014), although higher methylation of younger elements was not a feature of LTRs or LINEs.

We show that hypomethylation of HERVs is a generalized phenomenon in that ERVK, ERVL, and ERVL-MaLR elements all show 33% relatively lower methylation in the placenta, although ERV1-containing fragments are only 24% less methylated. As a consequence of hypomethylation, the placenta expresses more HERV-LTR-derived sequences than any other tissue (Reiss *et al.* 2007). The most well studied hypomethylated HERV-derived gene in the human placenta is *ERVWE1* (the gene for Syncytin-1) (Mi *et al.* 2000), which plays an essential role in cytotrophoblast fusion during syncytium formation (Blond *et al.* 2000). We and others previously found the LTR-derived *ERVWE1* promoter to exhibit placental-specific hypomethylation (Matouskova *et al.* 2006; Macaulay *et al.* 2011). Importantly, inhibition of *ERVWE1* results in a substantial decrease in cytotrophoblast fusion and differentiation *in vitro* (Frendo *et al.* 2003), while knockout of the murine syncytin genes is associated with defective placental structures and developmental lethality (Dupressoir *et al.* 2011). In addition, numerous other retrovirus-derived genes are specifically expressed in the human placenta pointing to other, as yet known, functional consequences of hypomethylation. These include *KCNH5* (Alu), *INSL4* (HERV-LTR), *EDNRB* (HERV-LTR), *PTN* (HERV-LTR), *MID1* (HERV-LTR), *IL2RB* (HERV-LTR), and *PEG10* [Ty3/Gypsy family LTR] (Ono *et al.* 2006; Cohen *et al.* 2011; Macaulay *et al.* 2011).

Contrary to our hypothesis that hypomethylation is specific to retroelements, we observed more frequent hypomethylation of nonretrotransposon-containing fragments, providing support for alternative hypotheses. For example, marked hypomethylation occurs in satellite repeats including pericentromeric repeats (Table S4) (Gama-Sosa *et al.* 1983) and is likely to contribute to genomic instability (Tsien *et al.* 2002) which, as previously hypothesized, may mitigate the risk of invasive trophoblastic disease (Hemberger 2010). Benefits of hypomethylation may also be obtained through expression of microRNAs, especially those from the C19MC cluster (Noguer-Dance *et al.* 2010) that confer resistance of trophoblasts and nonplacental cells to infection by DNA and RNA viruses (Delorme-Axford *et al.* 2013).

Our gene ontology enrichment analysis extends the functions of placentally-hypomethylated genes to include those involved in cell adhesion and apoptosis. During placental development, cell adhesion molecules on the surface of the placental trophoblast mediate its invasion into the uterine tissue (Damsky *et al.* 1992), especially through the expression of vascular cell adhesion receptors on the trophoblast (Zhou *et al.* 1997). The enrichment of genes involved in the regulation of apoptosis may be relevant to the apoptosis of endovascular trophoblasts that is important for the proper transformation of maternal arteries during placental development (Huppertz and Herrler 2005).

Our observation of deeper hypomethylation in nonretrotransposon-containing fragments does not, however, necessitate that these sequences are the functional target of hypomethylation in the placenta. Global hypomethylation may be required to achieve hypomethylation of the true targets, the functional retrogenes, in the face of mechanisms that maintain methylation-induced silencing of many retroelements or that suppress retrotransposon activity such as genome-defense genes, including *Tex19.1*, which suppresses L1 retrotransposons in the placenta (Reichmann *et al.* 2013).

In summary, comprehensive profiling of placental compared to neutrophil methylation shows a higher degree of placental hypomethylation in nonretrotransposon elements compared to retrotransposons. Our findings imply that retrotransposon expression might not be the evolutionary driver of placental hypomethylation. Future work on placental hypomethylation will need to carefully consider the implications of nonretroelement-associated sequences on placental function.

## Supplementary Material

Supplemental Material
