# Supplementary material for: Placental Hypomethylation Is More Pronounced in Genomic Loci Devoid of Retroelements
Source: G3 (Bethesda). 2016 Apr 27;6(7):1911–21. doi: 10.1534/g3.116.030379 (PMC4938645; doi:10.1534/g3.116.030379)
Supplement: Supplemental Material [file supp_6_7_1911__index.html]

Placental Hypomethylation Is More Pronounced in Genomic Loci Devoid of Retroelements — Supplemental Material 

# Placental Hypomethylation Is More Pronounced in Genomic Loci Devoid of Retroelements

## Supplemental Material for Chatterjee *et al.*, 2016

**Files in this Data Supplement:**

- Figure S1 - Methylation of human neutrophils showing all 279,762 *total analysed fragments*. (.pdf, 115 KB)
- Table S8 - Overlap of placental hypomethylated DMFs with placental partially methylated domains (PMDs). (.pdf, 38 KB)
- Table S9 - Gene ontology enrichment of genes containing hypomethylated placenta DMFs. (.pdf, 45 KB)
- Figure S2 - Methylation of human placenta showing all 279,762 *total analysed fragments*. (.pdf, 123 KB)
- Table S10 - Gene ontology enrichment of genes containing hypermethylated placenta DMFs. (.pdf, 46 KB)
- Figure S3 - Chromosome-wise methylation of human neutrophils. (.pdf, 472 KB)
- Figure S4 - Chromosome-wise methylation plot of human placenta. (.pdf, 649 KB)
- Figure S5 - Number of CpG sites per fragment vs. methylation for neutrophils. (.pdf, 305 KB)
- Figure S6 - Number of CpG sites per fragment vs. methylation for placenta. (.pdf, 336 KB)
- Figure S7 - Relationship of gene density and hypomethylated DMFs. (.pdf, 114 KB)
- Figure S8 - Relationship of gene density and hypermethylated DMFs. (.pdf, 92 KB)
- Figure S10 - Relationship of CpG density and hypermethylated DMFs. (.pdf, 95 KB)
- Figure S9 - Relationship of CpG density and hypomethylated DMFs. (.pdf, 118 KB)
- Table S1 - Summary of studies reporting placental methylation compared to somatic tissues. (.pdf, 43 KB)
- Table S2 - Clinical details of placental samples used in this study. (.pdf, 49 KB)
- Table S3 - Average methylation of major genomic elements and contribution to placental hypomethylation. (.pdf, 41 KB)
- Table S4 - Mean and median fragment methylation for each repeat element class in neutrophils and placenta. (.pdf, 50 KB)
- Table S5 - Methylation of retro vs. non-retroelement-containing fragments in neutrophils and placenta. (.pdf, 49 KB)
- Table S6 - Categories of retroelements that are highly methylated (>0.7) in neutrophils that retain very high methylation (> 0.8) in the placenta. (.pdf, 50 KB)
- Table S7 - Differentially methylated fragments in neutrophils and placenta. (.pdf, 52 KB)
- File S1 - Excel spread sheet containing information on 6146 differentially methylated fragments (DMFs) identified in the present study that were hypomethylated in human placenta. (.xlsx, 707 KB)
