# Supplementary material for: Placental Hypomethylation Is More Pronounced in Genomic Loci Devoid of Retroelements
Source: G3 (Bethesda). 2016 Apr 27;6(7):1911–21. doi: 10.1534/g3.116.030379 (PMC4938645; doi:10.1534/g3.116.030379)
Supplement: Supplemental Material [file supp_g3.116.030379_TableS8.pdf]

Table S8: Overlap of placental hypomethylated DMFs with placental partially methylated domains (PMDs).

Number of fragments analysed	Number of fragments in PMD regions	Percentage overlapping
32163 (comparison fragments)	8783	27.3
26017 (placental non-hypo fragments)	6494	25.0
6146 (hypomethylated in placenta)	2289	37.2
