# Supplementary material for: Placental Hypomethylation Is More Pronounced in Genomic Loci Devoid of Retroelements
Source: G3 (Bethesda). 2016 Apr 27;6(7):1911–21. doi: 10.1534/g3.116.030379 (PMC4938645; doi:10.1534/g3.116.030379)
Supplement: Supplemental Material [file supp_g3.116.030379_TableS9.pdf]

Table S9. Gene ontology enrichment of genes containing hypomethylated placenta DMFs[1]

Annotation term	Number of genes	Fold enrichment[2]	P value[3]
**Biological Process**			
Epithelial structure maintenance	3	29.4	0.004
Tissue homeostasis	6	5.6	0.004
Digestion	6	3.9	0.019
Visual perception	9	2.5	0.030
Negative regulation of cell differentiation	9	2.5	0.030
Cellular amino acid biosynthetic process	4	4.6	0.055
Regulation of cell projection organization	5	3.3	0.064
Enzyme linked receptor protein signaling pathway	11	1.9	0.066
Regulation of mitosis	4	4.2	0.069
Negative regulation of granulocyte differentiation	2	23.5	0.082
Protein complex assembly	14	1.6	0.088
ncRNA metabolic process	8	2.0	0.096
Blood circulation	7	2.2	0.096
**Molecular Function**			
Enzyme inhibitor activity	10	2.2	0.041
Ras guanyl-nucleotide exchange factor activity	5	3.3	0.062
Structural molecule activity	17	1.6	0.073
Oxidoreductase activity, acting on the CH-NH group of donors	3	6.1	0.086
Protein C-terminus binding	6	2.5	0.092
Alkali metal ion binding	8	2.1	0.093
**Cellular Component**			
Anchored to membrane	10	2.6	0.015
Extracellular matrix	13	2.2	0.017
Cilium	6	2.7	0.074
Plasma membrane part	47	1.2	0.099
Intermediate filament	7	2.2	0.100

[1] 405 DMFs hypomethylated in placenta overlapped the promoters of 355 genes. DAVID was used for gene ontology enrichment of 323 unique gene IDs.
[2] Fold enrichment of gene ontology annotations compared to background of all human protein-coding genes.
[3] DAVID uses a modified Fisher's exact test for enrichment evaluation analysis.
