# Supplementary material for: Placental Hypomethylation Is More Pronounced in Genomic Loci Devoid of Retroelements
Source: G3 (Bethesda). 2016 Apr 27;6(7):1911–21. doi: 10.1534/g3.116.030379 (PMC4938645; doi:10.1534/g3.116.030379)
Supplement: Supplemental Material [file supp_g3.116.030379_FigureS2.pdf]

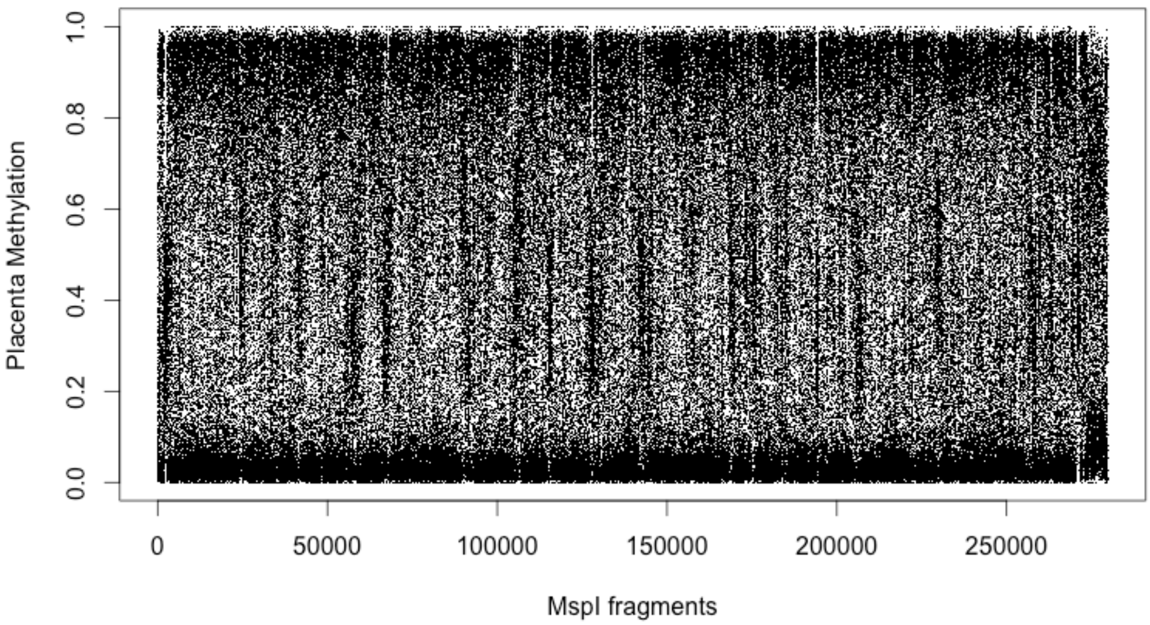

Figure S2. Methylation of human placenta showing all 279,762 *total analysed fragments*. The x-axis shows 279,762 fragments where high quality information was available from at least one individual, and the y-axis shows the mean methylation (scale of 0 to 1.0).
