# Supplementary material for: Placental Hypomethylation Is More Pronounced in Genomic Loci Devoid of Retroelements
Source: G3 (Bethesda). 2016 Apr 27;6(7):1911–21. doi: 10.1534/g3.116.030379 (PMC4938645; doi:10.1534/g3.116.030379)
Supplement: Supplemental Material [file supp_g3.116.030379_TableS10.pdf]

Table S10. Gene ontology enrichment of genes containing hypermethylated placenta DMFs[1]

Annotation term	Number of genes	Fold enrichment[2]	P value[3]
**Biological Process**			
Positive regulation of transcription	10	3.9	0.001
Gut morphogenesis	3	38.5	0.003
Mesoderm development	4	11.8	0.005
Blood vessel development	6	5.3	0.005
Morphogenesis of an epithelium	4	8.6	0.011
Regulation of T cell differentiation	3	12.8	0.022
Regulation of cell proliferation	9	2.5	0.024
Macrophage differentiation	2	54.5	0.036
Gastrulation	3	9.4	0.040
Chordate embryonic development	5	3.3	0.062
Macrophage activation	2	29.1	0.066
Regulation of programmed cell death	8	2.1	0.072
Limb morphogenesis	3	6.6	0.073
Negative regulation of cell differentiation	4	4.0	0.074
Respiratory tube development	3	6.4	0.077
Female pregnancy	3	6.0	0.088
**Molecular Function**			
Transcription regulator activity	20	3.2	3.9×10^{-6}
Identical protein binding	8	3.0	0.015

[1] 107 DMFs hypermethylated in placenta overlapped the promoters of 98 genes. DAVID was used for gene ontology enrichment of 88 unique gene IDs.
[2] Fold enrichment of gene ontology annotations compared to background of all human protein-coding genes.
[3] DAVID uses a modified Fisher's exact test for enrichment evaluation analysis.
