# Supplementary material for: Placental Hypomethylation Is More Pronounced in Genomic Loci Devoid of Retroelements
Source: G3 (Bethesda). 2016 Apr 27;6(7):1911–21. doi: 10.1534/g3.116.030379 (PMC4938645; doi:10.1534/g3.116.030379)
Supplement: Supplemental Material [file supp_g3.116.030379_FigureS4.pdf]

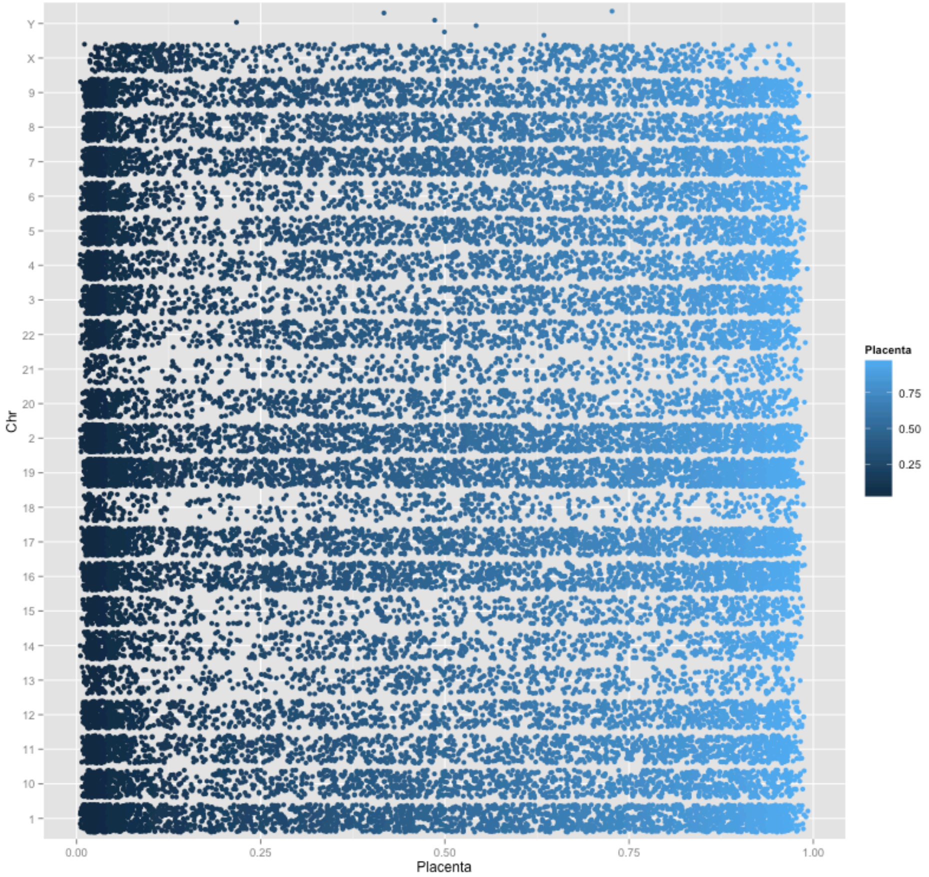

Figure S4. Chromosome-wise methylation plot of human placenta. The y-axis shows chromosomes and x-axis shows methylation of each of the *comparison fragments* (scale of 0 to 1.0). Note: the data shown here are the same as in Figure 1b, but in Fig S4 they are separated by chromosome.
