# Supplementary material for: Placental Hypomethylation Is More Pronounced in Genomic Loci Devoid of Retroelements
Source: G3 (Bethesda). 2016 Apr 27;6(7):1911–21. doi: 10.1534/g3.116.030379 (PMC4938645; doi:10.1534/g3.116.030379)
Supplement: Supplemental Material [file supp_g3.116.030379_FigureS6.pdf]

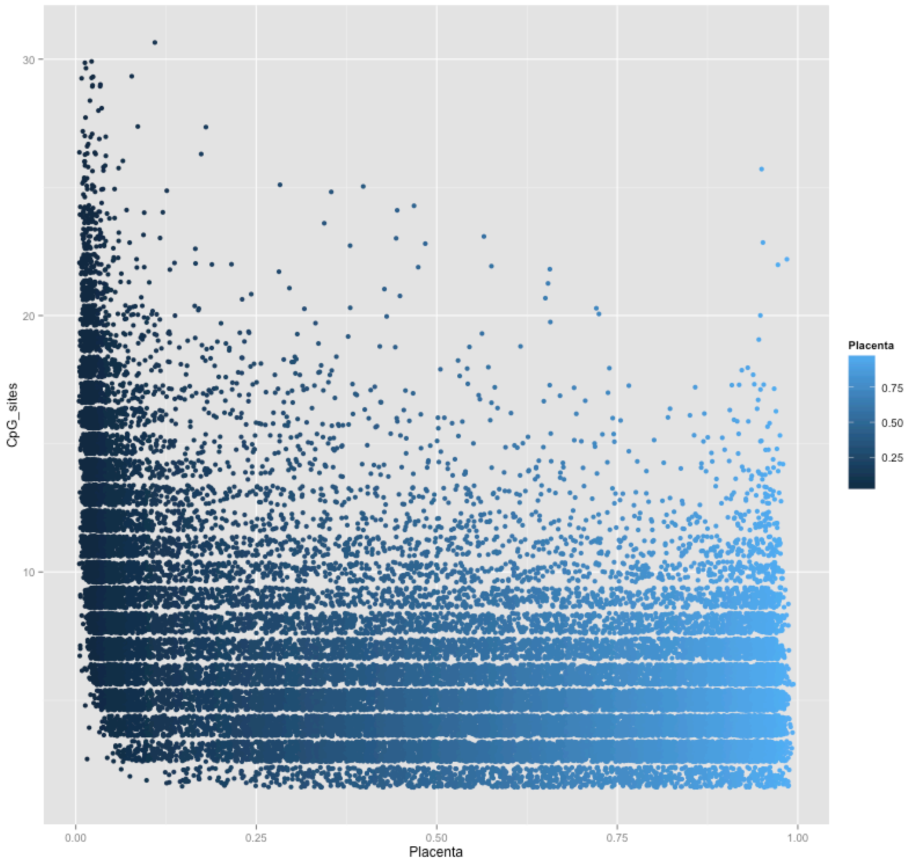

Figure S6. Number of CpG sites per fragment vs. methylation for placenta. The y-axis shows the number of CpG sites in each fragment. The x-axis shows the mean methylation value for that fragment (scale of 0 to 1.0).
