# Supplementary material for: Placental Hypomethylation Is More Pronounced in Genomic Loci Devoid of Retroelements
Source: G3 (Bethesda). 2016 Apr 27;6(7):1911–21. doi: 10.1534/g3.116.030379 (PMC4938645; doi:10.1534/g3.116.030379)
Supplement: Supplemental Material [file supp_g3.116.030379_FigureS7.pdf]

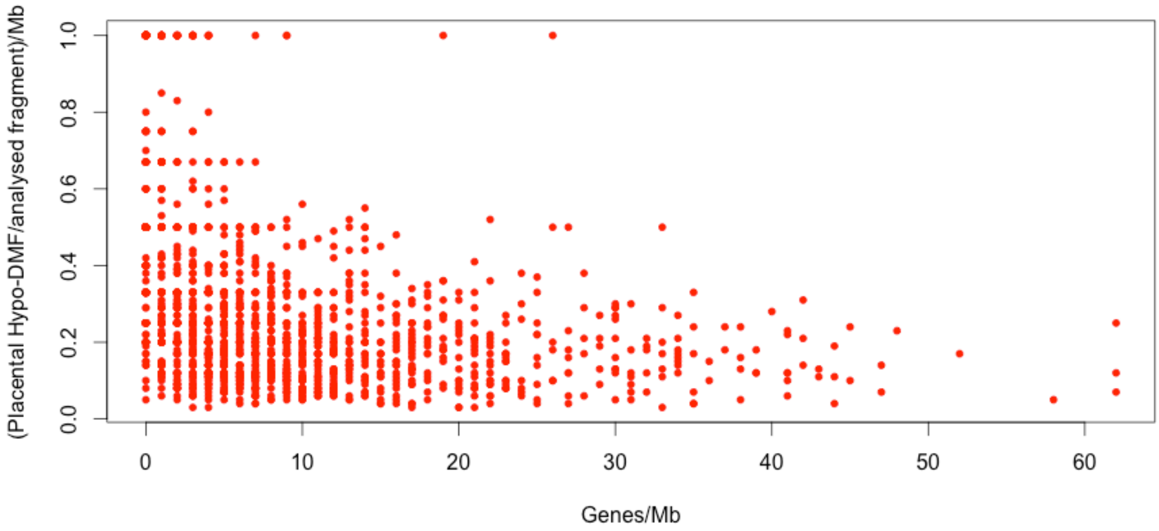

Figure S7. Relationship of gene density and hypomethylated DMFs. The y-axis shows the DMF density score, which was generated by dividing the number of DMFs by the number of analysed fragments for each 1 Mb window across the human genome. Tiling windows with at least three analysed fragments were considered for this analysis. The x-axis shows the number of protein coding genes in a 1 Mb window.
