# Supplementary material for: Placental Hypomethylation Is More Pronounced in Genomic Loci Devoid of Retroelements
Source: G3 (Bethesda). 2016 Apr 27;6(7):1911–21. doi: 10.1534/g3.116.030379 (PMC4938645; doi:10.1534/g3.116.030379)
Supplement: Supplemental Material [file supp_g3.116.030379_TableS1.pdf]

Table S1. Summary of studies reporting placental methylation compared to somatic tissues.

Study	Method	Somatic	Placenta	%hypomethyla-tion of placenta	Type of somatic cells
Ehrlich 1982 (9)	HPLC %mC of total bases	0.93	0.76	18%	Heart, liver, lungs, spleen, lymphocytes, brain, thymus
Tsein 2002 (10)	HPLC %methyl-ation of Cs	3.72	3.20	14%	Cerebellum, spleen, lung, heart, liver
Fuke 2004 (11)	HPLC %methyl-ation of Cs	3.99	2.99	25%	Whole blood
Novakovic 2010 (12)	HPLC %methyl-ation of Cs	3.82	2.93	25%	Cord blood
Schroeder 2013 (13)	WGBS %methyl-ation at CpGs	76.72	62.92	18%	cerebral cortex, cerebellum, NK cells, kidney
Current study	RRBS %methyl-ation at CpGs	47.50	37.10	22%	Neutrophils

HPLC, high performance liquid chromatography
WGBS, whole genome bisulphite sequencing
RRBS, reduced representation bisulphite sequencing
