# Supplementary material for: Placental Hypomethylation Is More Pronounced in Genomic Loci Devoid of Retroelements
Source: G3 (Bethesda). 2016 Apr 27;6(7):1911–21. doi: 10.1534/g3.116.030379 (PMC4938645; doi:10.1534/g3.116.030379)
Supplement: Supplemental Material [file supp_g3.116.030379_TableS2.pdf]

Table S2. Clinical details of placental samples used in this study.

Study number	Gest	Mat Age	Mat BMI	Baby BW (g)	Placental W (g)	Sex	Smoker	Parity
88	24.2	29	30.5	825	155	m	n	G2P1
47	30.3	28	23	1475	395	m	n	G2P0
46	30.3	28	23	1275	395	f	n	G2P0
159	33.4	19	25.9	2000	405	f	n	G4P2
140	35.2	32	27.3	3120	538	f	y	G1P0
121	36.1	30	30.1	2880	504	f	n	G1P0
122	36.6	39	27.7	3490	526	m	n	G1P0
478	38.1	33	22.9	3100	464	m	n	G1P0
410	39.1	35	33.5	3965	452	f	n	G2P0
32	39.3	32	25.4	2870	370	f	n	G1P1
416	40.2	42	19.7	4040	590	m	n	G7P3

Abbreviations: Gest, gestation; Mat, maternal; BMI, body mass index; BW, birth weight; W, weight
