# Supplementary material for: Placental Hypomethylation Is More Pronounced in Genomic Loci Devoid of Retroelements
Source: G3 (Bethesda). 2016 Apr 27;6(7):1911–21. doi: 10.1534/g3.116.030379 (PMC4938645; doi:10.1534/g3.116.030379)
Supplement: Supplemental Material [file supp_g3.116.030379_TableS3.pdf]

Table S3: Average methylation of major genomic elements and contribution to placental hypomethylation.

	NT (mean)	PL (mean)	Number of fragments	Difference in methylation	Weighted contribution to decreased methylation
Promoter -5 - +1 kb	0.20	0.17	6109	0.03	0.57
Intergenic (> 5 kb upstream)	0.70	0.51	12254	0.19	7.2
Exon	0.71	0.59	2000	0.12	0.75
Intron	0.72	0.58	10211	0.14	4.5
Gene body boundary	0.74	0.60	1543	0.14	0.67
All repeats	0.75	0.58	11188	0.17	5.9

Abbreviations: NT: neutrophils; PL: placenta
