# Supplementary material for: Placental Hypomethylation Is More Pronounced in Genomic Loci Devoid of Retroelements
Source: G3 (Bethesda). 2016 Apr 27;6(7):1911–21. doi: 10.1534/g3.116.030379 (PMC4938645; doi:10.1534/g3.116.030379)
Supplement: Supplemental Material [file supp_g3.116.030379_TableS4.pdf]

Table S4: Mean and median fragment methylation for each repeat element class in neutrophils and placenta.

			Mean		Median		
Repeat classes	Sub class	Number. of fragments	NT	PL	NT	PL	Difference
SINE	Alu	4577	0.878	0.682	0.940	0.759	0.18
SINE	MIR	735	0.730	0.549	0.892	0.575	0.32
LINE	L1	612	0.833	0.620	0.924	0.651	0.27
LINE	L2	529	0.789	0.597	0.910	0.635	0.28
LTR	ERV1	1215	0.868	0.672	0.929	0.709	0.22
LTR	ERVK	121	0.791	0.550	0.904	0.594	0.31
LTR	ERVL	258	0.827	0.602	0.912	0.605	0.31
LTR	ERVL-MaLR	360	0.866	0.613	0.922	0.616	0.31
Satellite	Satellite, centromeric, telomeric	125	0.796	0.522	0.878	0.529	0.35
Low complexity	Low complexity	1138	0.095	0.099	0.025	0.032	-0.01
Simple repeat	Simple repeat	253	0.207	0.169	0.025	0.037	-0.01
DNA repeat	DNA repeat	420	0.797	0.598	0.914	0.630	0.28
Other repeat	Other repeat	116	0.928	0.819	0.947	0.834	0.11

Abbreviations: NT: neutrophils; PL: placenta
