# Supplementary material for: Placental Hypomethylation Is More Pronounced in Genomic Loci Devoid of Retroelements
Source: G3 (Bethesda). 2016 Apr 27;6(7):1911–21. doi: 10.1534/g3.116.030379 (PMC4938645; doi:10.1534/g3.116.030379)
Supplement: Supplemental Material [file supp_g3.116.030379_TableS5.pdf]

Table S5: Methylation of retro vs. non-retroelement-containing fragments in neutrophils and placenta.

	Retroelements						Non-retroelements					
		Mean		Median				Mean		Median		
Elements	# of fragments	NT	PL	NT	PL	Difference	# of fragments	NT	PL	NT	PL	Difference
Promoter	680	0.627	0.460	0.863	0.446	0.416	5401	0.146	0.131	0.033	0.043	-0.010
Intergenic	4664	0.863	0.632	0.927	0.660	0.267	7376	0.594	0.424	0.816	0.416	0.400
Exon	39	0.821	0.705	0.923	0.788	0.136	1922	0.710	0.589	0.924	0.689	0.235
Intron	3159	0.861	0.704	0.943	0.800	0.143	8387	0.550	0.437	0.790	0.408	0.382

Abbreviations: NT, neutrophils; PL, placenta
