# Supplementary material for: Placental Hypomethylation Is More Pronounced in Genomic Loci Devoid of Retroelements
Source: G3 (Bethesda). 2016 Apr 27;6(7):1911–21. doi: 10.1534/g3.116.030379 (PMC4938645; doi:10.1534/g3.116.030379)
Supplement: Supplemental Material [file supp_g3.116.030379_TableS6.pdf]

Table S6. Categories of retroelements that are highly methylated (>0.7) in neutrophils that retain very high methylation (> 0.8) in the placenta. Columns 2 and 3 show the composition of the retrotransposons that retain or do not retain high methylation. The percentage of each class that retains high methylation in the placenta (> 0.8) is shown in column 4.

Total	Plac > 0.8	Plac < 0.8	Percent of category that retains methylation > 0.8 in placenta
L1	0.059	0.096	24.0%
L2	0.039	0.051	28.4%
Alu	0.558	0.452	38.6%
MIR	0.040	0.066	23.6%
ERVK	0.008	0.015	21.8%
ERVL	0.023	0.041	22.1%
ERVL-MaLR	0.034	0.058	23.1%
ERV1	0.211	0.195	35.5%
All retroelements	1.000	1.000	
