# Supplementary material for: Placental Hypomethylation Is More Pronounced in Genomic Loci Devoid of Retroelements
Source: G3 (Bethesda). 2016 Apr 27;6(7):1911–21. doi: 10.1534/g3.116.030379 (PMC4938645; doi:10.1534/g3.116.030379)
Supplement: Supplemental Material [file supp_g3.116.030379_TableS7.pdf]

Table S7. Differentially methylated fragments in neutrophils and placenta.

**Criteria**	**R≥6, S≥6***
Significant fragments ($1.55\times10^{-6**}$ and > 0.25 diff)	6767
CpG sites within these	38307
Mean CpGs per fragment	5.66
Placenta hypo	6146
Mean CpGs per fragment (hypo)	5.56
Placenta hyper	621
Mean CpGs per fragment (hyper)	6.67
% of DMFs that are hypo meth in placenta	90.8

* R = number of neutrophil samples fulfilling coverage criteria and S = number of placenta samples fulfilling coverage criteria.
** Significance level = 0.05, adjusted with multiple test correction by the Bonferroni method.
